# Optical diffraction tomographic microscopy: a cutting-edge label-free three-dimensional bioimaging

**DOI:** 10.52601/bpr.2025.250026

**Published:** 2026-08-31

**Authors:** Junwei Min, Peng Gao, Xun Yuan, Yuge Xue, Ruihua Liu, Yingjie Feng, Siying Wang, Yan Li, Kai Wen, Liming Yang, Tengfei Wu, Baoli Yao

**Affiliations:** 1 State Key Laboratory of Ultrafast Optical Science and Technology, Xi’an Institute of Optics and Precision Mechanics, Chinese Academy of Sciences, Xi’an 710119, China; 2 School of Physics, Xidian University, Xi’an 710071, China; 3 Guangzhou National Laboratory, Guangzhou 510005, China; 4 University of Chinese Academy of Sciences, Beijing 100049, China

**Keywords:** Optical diffraction tomography, Optical microscopy, Quantitative phase imaging, Tomographic imaging, Refractive index measurement, Three-dimensional data reconstruction

## Abstract

Optical diffraction tomographic microscopy (ODTM) is an advanced label-free three-dimensional optical microscopic imaging technique. It measures the three-dimensional refractive index (RI) distributions of unstained, transparent biological specimens with high resolution from scattered fields based on the diffraction tomography theorem. Both the morphological and biophysical parameters, as well as the internal organelles of the specimen, can be further analyzed from the measured RI values. ODTM has been increasingly employed in the field of biology, yielding numerous promising results. In order to further promote the application and popularization of this technology in biological research, we provide a tutorial on the fundamental principles and instrumentation of ODTM. The distinct characteristics of ODTM using various illumination strategies and reconstruction algorithms are presented. Observation results from single cells, tissues, and small-scale biological objects are shown to demonstrate the superior performance of ODTM. Current trends and future perspectives of ODTM are discussed.

## INTRODUCTION

Since Robert Hooke's pioneering observation of cork cells through a microscope in the 17th century, optical microscopy has evolved into an indispensable imaging tool in biology. Because most biological specimens are optically transparent and exhibit weak scattering and absorption properties, they yield low-contrast images in brightfield microscopy. Various microscopic techniques have been developed to achieve better imaging capabilities. The invention of phase-contrast microscopy (PCM) (Zernike 1942) and differential interference contrast microscopy (DICM) (Allen *et al.* 1969) initiated the first revolution in biological imaging by successfully converting phase information into high contrast intensity variations. This substantial improvement in contrast enabled the visualization of previously indiscernible structures. The second revolution in optical microscopy was initiated with the discovery of the Green Fluorescent Protein and its application as a fluorescent marker (Shimomura 2005). Through the specific labeling of target molecules using fluorescent probes, unparalleled molecular specificity and imaging contrast could be achieved. Although fluorescence microscopic techniques are appreciated, fluorescent markers may induce unfavorable effects like phototoxicity and photobleaching, and they do not allow long time investigation and overall imaging of the specimen. As a consequence, label-free imaging modes are regaining more and more attention, as there are no phototoxic effects associated with staining/labelling, and permit essentially unlimited observation time (Liu *et al.* 2000; Shribak and Inoué 2006).

In both PCM and DICM, the image is formed by a complex interaction of the incoherent illuminating light with the specimen. The recorded image qualitatively reveals the profiles of the observed specimen, but does not deliver quantitative information. To quantitatively characterize biological specimens, a variety of quantitative phase imaging (QPI) techniques have been proposed, including but not limited to digital holographic microscopy (DHM) (Marquet *et al.* 2005), spatial light interference interferometry (SLIM) (Wang *et al.* 2011a), diffraction phase microscopy (DPM) (Bhaduri *et al.* 2014), quadriwave lateral shearing interferometry (QLSI) (Bon *et al.* 2009), transport of intensity equations (Zuo *et al.* 2020), Fourier ptychography (Ou *et al.* 2013) and so forth (Bu *et al.* 2024; Li *et al.* 2019; Lue *et al.* 2007; Tian and Waller 2015; Yamaguchi and Zhang 1997). QPI measures the optical phase delay (OPD) resulting from the refractive index (RI) discrepancy between a specimen and its surrounding medium. Since the RI is an intrinsic optical property of a material, no external labeling agents are required to generate imaging contrast in QPI. Furthermore, the morphological and chemical properties of a specimen can be quantitatively analyzed through the RI distribution. These advantages make QPI open the door for direct analysis of live cells and their pathophysiological alterations (Nguyen *et al.* 2022). Combined with improved optical designs and computational algorithms, QPI has played a rapidly expanding role in biomedical applications over the past decades, such as quantifying the shape and dynamics of living cells in their native state (Bettenworth *et al.* 2014; Kemper and von Bally 2008; Popescu *et al.* 2006), optical metrology of nanostructures (Anand *et al.* 2022; Coppola *et al.* 2004; Gao *et al.* 2021; Xu *et al.* 2022), and drug release monitoring *in vitro* (Gabai *et al.* 2013).

Although QPI techniques present new opportunities for studying cells and tissues non-invasively and quantitatively, they are not truly three-dimensional (3D) imaging techniques in the sense that volumetric sample information is not accessible, as OPD is a product of thickness and RI difference in the axial direction. In order to obtain more precise morphological specifications, such as nucleus shape, dry mass, and nucleus-to-cytoplasm volume ratio, depth-resolved, tomographic imaging is required. Subsequently, optical diffraction tomographic microscopy (ODTM) has undergone intensive research (Wedberg and Stamnes 1995). ODTM is also termed as synthetic aperture microscopy (Nahamoo *et al.* 1984), tomographic diffractive microscopy (TDM) (Haeberlé *et al.* 2010), tomographic phase microscopy (TPM) (Debailleul *et al.* 2008; Jin *et al.* 2017), holographic tomography (HT) (Villone *et al.* 2018), or holotomography (Kim *et al.* 2024a; Kujawińska *et al.* 2019). It enables volumetric imaging of biological samples by mapping their 3D RI distribution from measured multiple two-dimensional optical fields based on the Fourier diffraction theorem. Since the RI value of most biological specimens is linearly proportional to protein concentration, the measured RI values can be further translated into various useful parameters such as protein concentration and cellular dry mass (Barer 1952; Kim *et al.* 2017; Popescu 2011).

ODTM was first theoretically proposed in 1969 by E. Wolf (Wolf 1969), in which he proposed the Fourier diffraction theorem using the Born approximation, which attempts to reconstruct the spatial distribution of the 3D object by sequentially illuminating the sample with tilted incident fields. This concept was later given a geometrical interpretation by Dändliker and Weiss (Dändliker and Weiss 1970). However, owing to the constraints imposed by the experimental conditions and technological limitations at that time, the concept was not applied to ODTM for three-dimensional cellular imaging. Thanks to the advancements in laser sources, detecting devices, and computing powers, ODTM has regained interest. In 2006 and 2007, two groups reported the implementations of ODTM for 3D imaging of cells by quantitatively mapping their RI distributions (Charrière *et al.* 2006; Choi *et al.* 2007). In these early works, the ODTM systems were based on sample rotations with fixed laser illumination or scanning of laser illumination angle with the help of galvanometer mirrors (GM) to obtain multiple two-dimensional optical fields, and adopted the filtered back-projection (FBP) method to reconstruct 3D RI distribution. However, GM introduced unavoidable mechanical instability, and sample rotation may destroy the original state of the sample. Additionally, the FBP method ignored the optical diffraction effect, and this inaccurate tomography model significantly reduces the 3D reconstruction accuracy for cells. In 2009, a Rytov approximation model was introduced into ODTM, which obtained a higher accuracy of 3D reconstruction of live cells than before (Sung *et al.* 2009). Since then, numerous research teams have refined the ODTM technique. In terms of hardware improvement, spatial light modulator (SLM), digital micromirror device (DMD), super-continuum laser, programmable LED array, fast camera, and graphics-processing unit (GPU) have been integrated into ODTM systems to enhance theirs imaging stability, quality, and speed. Furthermore, the employment of diverse QPI methods to acquire multiple optical fields, including both interferometric and non-interferometric methods, leads to varied structural configurations within ODTM systems. These distinct imaging setups consequently result in differing imaging characteristics. In terms of software improvement, diffraction reconstruction algorithms have been proposed to improve the reconstruction accuracy for the cells and other small samples RI variations over the wavelength scale. Advanced image processing tools, including total variation regularization, deconvolution algorithms, and machine learning, also have been applied to ODTM to overcome the hardware and physical limits such as missing cone and missing apple core problems (Jo *et al.* 2021). With its quantitative, label-free, three-dimensional tomographic imaging characteristics, and high spatiotemporal resolution features, ODTM has emerged as a powerful tool for histopathology (Kim *et al.* 2016b, 2024b; Lee *et al.* 2024; Li *et al.* 2025; Yang *et al.* 2017), hematology (Jung *et al.* 2016b; Lee *et al.* 2016; Yoon *et al.* 2015; Zhao *et al.* 2022), microbiology (Jung *et al.* 2018; Lee *et al.* 2022a), cell biology (Anantha *et al.* 2023; Dubois *et al.* 2006; Kim *et al.* 2016a) and nanotechnology (Kang *et al.* 2023; Kim *et al.* 2018).

ODTM gathers many advantages, but is still far from being the most popular three-dimensional label-free quantitative optical imaging tool for biology research, as most researchers lack sufficient understanding of the principle and performance of this technology. To promote the dissemination of the ODTM technique and enhance its accessibility and utility in practical biological research, the principle of ODMT is reviewed, and a detailed analysis of the instrumental requirements and performance characteristics of distinct ODMT systems is provided. Recent advances in the development of ODTM techniques are introduced, and some classical applications of ODTM in the fields of biology and medicine are presented. Finally, perspectives of ODTM on potential future developments and applications are discussed.

## PRINCIPLE OF ODTM

In this section, we briefly introduce Wolf’s original theory and Devaney’s modification to adopt the Rytov approximation. A more detailed introduction to the fundamentals of diffraction and tomographic microscopy within the framework of the Born approximation is referred to the references (Balasubramani *et al.* 2021; Devaney 1981; Wolf 1969).

**Figure 1:**
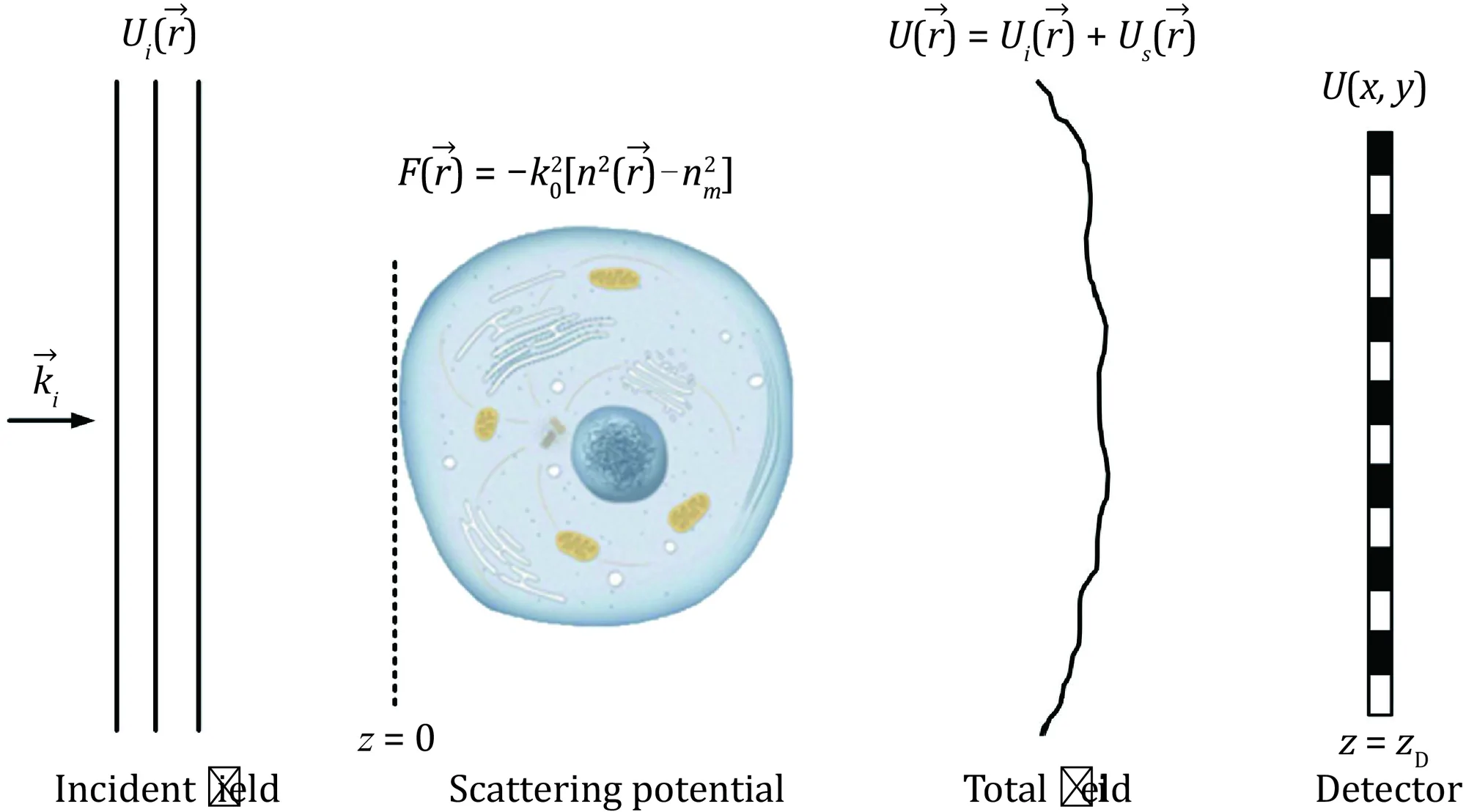
Schematic of light wave propagation through a weakly scattering object. The monochromatic plane wave *U*_*i*_($\vec {\boldsymbol{r}}  $) passes through a weakly scattering object to form the total field *U*($\vec {\boldsymbol{r}}  $) that carries the scattering potential *F*$\vec {\boldsymbol{r}}  $) information of the object. The projection of the total field *U*(*x*, *y*) at the imaging plane z = z_D_ is detected for reversed reconstruction. Adapted from Ref. Yuan *et al.* (2024)

Assuming a weakly scattering object with a RI distribution *n*($\vec {\boldsymbol{r}} $) is immersed in a medium with RI*n*_m_. With the scalar field assumption, when a plane monochromatic wave *U*_*i*_($\vec {\boldsymbol{r}} $) = exp($j \vec {\boldsymbol{k}}_i \cdot \vec {\boldsymbol{r}} $) with a wavevector of $\vec {\boldsymbol{k}}_i $= (*k*_*ix*_, *k*_*iy*_, *k*_*iz*_,) and wavelength *λ* passes through the object, as depicted in Fig. 1, the total output field *U*($\vec {\boldsymbol{r}} $) follows the Helmholtz equation:

1 $ {\nabla ^2}U(\vec {\boldsymbol{r}} ) + {k_0}^2n{(\vec {\boldsymbol{r}} )^2}U(\vec {\boldsymbol{r}} ) = 0 , $

here, $\vec {\boldsymbol{r}} $ = (*x*, *y*, *z*) is the spatial coordinate, $\nabla ^2 $ is the Laplace operator, *k*_0_ = 2π/*λ* is the wave number in vacuum. The wavevector in the immersion medium can be written as *k*_*i*_ = |$\vec {\boldsymbol{k}}_i $| = *k*_0_*n*_m_ = 2π*n*_m_/*λ*, and *k*($\vec {\boldsymbol{r}} $) = *k*_0_*n*($\vec {\boldsymbol{r}} $) denotes the wavevector of propagation in the object.

The incident field *U*_*i*_($\vec {\boldsymbol{r}} $) is a solution of the homogeneous Helmholtz equation, *i.e.,*

2 $ {\nabla ^2}{U_i}(\vec {\boldsymbol{r}} ) + {k_i}^2{U_i}(\vec {\boldsymbol{r}} ) = 0 . $

The total output field can be decomposed into the sum of the incident field ***U***_***i***_($\vec {\boldsymbol{r}} $) and the object scattered field *U*_*s*_($\vec {\boldsymbol{r}} $):

3 $ U ( \vec {\boldsymbol{r}} )= U _{ \boldsymbol{i} } ( \vec {\boldsymbol{r}} )+ U _{ \boldsymbol{s} } ( \vec {\boldsymbol{r}} ). $

Combining Eqs. 1, 2 and 3, the scattered field ***U***_***s***_($\vec {\boldsymbol{r}} $) satisfies the following equation:

4 $ \left( {{\nabla ^2} + {k_i}^2} \right){U_s}(\vec {\boldsymbol{r}} ) = - {k_0}^2[n{(\vec {\boldsymbol{r}} )^2} - {n_m}^2]U(\vec {\boldsymbol{r}} ) = F(\vec {\boldsymbol{r}} )U(\vec {\boldsymbol{r}} ) , $

where *F*($\vec {\boldsymbol{r}} $) = −*k*_0_^2^[*n*($\vec {\boldsymbol{r}} $)^2^ − *n*_m_^2^] is known as the object scattering potential function (or called object function), which is quantitatively related to the physical structure of the object. To quantitatively obtain *n*($\vec {\boldsymbol{r}} $), it should ascertain *F*($\vec {\boldsymbol{r}} $) based on the knowledge of the scattered field *U*_*s*_($\vec {\boldsymbol{r}} $).

Since the observed object is weakly scattering, *i.e.,* |***U***_***s***_($\vec {\boldsymbol{r}} $)| << |***U***_***i***_($\vec {\boldsymbol{r}} $)|, then *U*($\vec {\boldsymbol{r}} $) = *U*_*i*_($\vec {\boldsymbol{r}} $) + *U*_*s*_($\vec {\boldsymbol{r}} $)≈ *U*_*i*_($\vec {\boldsymbol{r}} $). Hence, a good approximation to the solution of Eq. 4 at the detection plan z = z_D_ for the scattered field under the illumination direction $\vec {\boldsymbol{k}}_i $ can be given by the first Born approximation:

5 \begin{equation*}\begin{split} &
{\left. {{U_{si}}(x,y)} \right|_{z = {z_D}}} \\&
= \frac{{ - j}}{{4\pi }}\iint {\frac{{{{\tilde F }_i}({k_{sx}} - {k_{ix}},{k_{sy}} - {k_{iy}},{k_{sz}} - {k_{iz}})}}{{{k_{sz}}}}{e^{j{k_{s{\mathrm{z}}}}{z_D}}}}\cdot \\&\quad
{e^{j({k_{sx}}x + {k_{sy}}y)}}d{k_{sx}}d{k_{sy}} ,
\end{split}\end{equation*}

where $ \vec {\boldsymbol{k}}_s $ = (*k*_*sx*_, *k*_*s*y_, *k*_*s*z_) is the scattered wavevector with the restriction of *k*_s*z*_= [*k*_*i*_^2^−*k*_s*x*_^2^−*k*_sy_^2^]^1/2^. $\tilde F_i (\vec K) $ is the 3D Fourier transform of the object function *F*($\vec {\boldsymbol{r}} $) under the illumination direction $\vec {\boldsymbol{k}}_i $. * \vec K * = (*K*_*x*_, *K*_y_, *K*_z_) is the object wavevector with the relationship of * \vec K * = $\vec {\boldsymbol{k}}_s $ −$\vec {\boldsymbol{k}}_i $*.* From Eq. 5, one can find that the scattered field *U*_*si*_($\vec {\boldsymbol{r}} $) is expressed as an angular spectrum of plane waves, weighted by the Fourier transform of the scattering potential *F*($\vec {\boldsymbol{r}} $). The term $\tilde F_i (\vec {\boldsymbol{k}}_s -\vec {\boldsymbol{k}}_i) $ indicates that the scattering of the object under a certain illumination angle is sensitive to the Fourier components of *F*($\vec {\boldsymbol{r}} $) at spatial frequency $\vec {\boldsymbol{k}}_s -\vec {\boldsymbol{k}}_i $.

By making the 2D Fourier transform on both sides of Eq. 5, one can obtain the following equation:

6 $ {\tilde F _i}(\vec K ) = \frac{{j{k_{sz}}}}{\pi }{e^{ - j{k_{sz}}{z_D}}} {\tilde U_{si}} ({k_{sx}},{k_{sy}}) . $

This is known as the Fourier diffraction theorem. Eq. 6 implies that a portion of the 3D Fourier components of the scattering potential can be determined from the knowledge of the 2D Fourier components of the scattered field in the detection plane. In practice, one can measure the incident field *U*_***i***_($\vec {\boldsymbol{r}} $) and total field *U*($\vec {\boldsymbol{r}} $) at the detection plane z = z_D_ for every illumination direction. Then the corresponding scattered field can be calculated as *U*_***si***_($\vec {\boldsymbol{r}} $)= *U*($\vec {\boldsymbol{r}} $)-*U*_***i***_($\vec {\boldsymbol{r}} $), and the corresponding portion of the 3D Fourier components of the scattering potential can be obtained based on the Fourier diffraction theorem Eq. 6. By varying the illumination direction $\vec {\boldsymbol{k}}_i $ and measuring all incident and total fields, the entire 3D spectrum of the scattering potential *\tilde F *(* \vec K *) = ∑$\tilde F_i $(* \vec K *) (∑ denotes sum operation for subscript *i*) can be mapped. Subsequently, making the inverse Fourier transform to *\tilde F *(* \vec K *), the 3D distribution of the object scattering potential function *F*($\vec {\boldsymbol{r}} $) can be quantitatively reconstructed. At last, the quantitative 3D RI distribution of the scattering object can be calculated:

7 $ n(\vec{r})=\sqrt{{n}_{m}^{2}-\left|IFT\left[{\displaystyle \sum {\tilde {F}}_{i}(\vec{K})}\right]\right|/{k}_{0}^{2}} , $

where *IFT* denotes the inverse Fourier transform operation.

**Figure 2:**
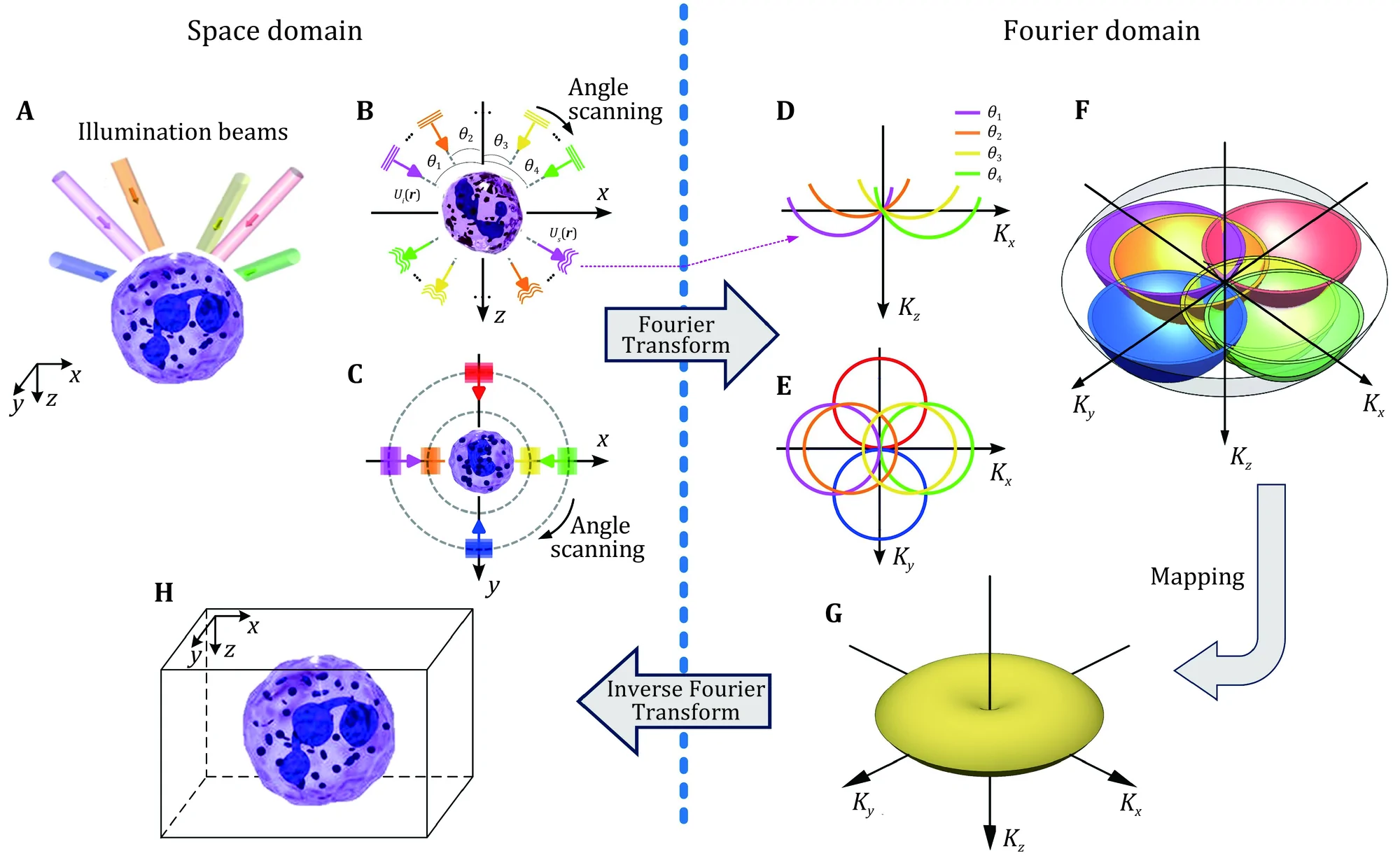
Principle and workflow chart of ODTM with rotated illumination angle. **A** A specimen is illuminated from different angles. **B**,**C** Cross-sectional slices of Panel A in the *x*−*z* plan and *x*−*y* plan, respectively. **D**,**E** Cross-sectional slices of the spectrum distribution in the *K*_*x*_−*K*_*z*_ plan and *K*_*x*_−*K*_*y*_ plan, respectively. **F** 3D spectrum distribution of scattering waves *U*_s_($\vec {\boldsymbol{r}} $) with different illumination angles. **G** Mapped 3D spectrum of *F*($\vec {\boldsymbol{r}} $). **H** Reconstructed 3D RI distribution *n*($\vec {\boldsymbol{r}} $) of the specimen

Figure 2 describes the principle and workflow chart of a common ODTM with the illumination angle rotated. When a specimen is sequentially illuminated by plane waves ***U***_*i*_($\vec {\boldsymbol{r}} $) from different angles, as shown in Fig. 2A, the projections of the corresponding scattering waves ***U***_*si*_($\vec {\boldsymbol{r}} $) can be measured at the detection plane. Figures 2B and 2C are the cross-sectional slices of illumination in the *x*−*z* and *x*−*y* plans, respectively. For a single illumination, only a portion of the Ewald sphere can be effectively collected because of the limited numerical aperture (NA) of the microscope objective, and it is located at a position related to the incident angle *θ*_*i*_, as shown in Fig. 2F. The cross-sectional slice of the Ewald sphere in the *K*_x_−*K*_z_ plan is a semi-circular arc, while in the *K*_x_−*K*_y_ plan is a circle arc as depicted in Figs. 2D and 2E, respectively. As a consequence, after illuminating the specimen with waves having different angles, different sets of Fourier components can be obtained, which then have to be correctly reassigned in the Fourier space to get the full 3D spectrum of the object function, as presented in Fig. 2G. Finally, by taking the inverse Fourier transform of it, the quantitative scattering potential ***F***($\vec {\boldsymbol{r}} $) of the specimen can be reconstructed, and then the 3D RI distribution *n*($\vec {\boldsymbol{r}} $) can be obtained as shown in Fig. 2H.

It should be noted that the numerical simulation has demonstrated that the Born approximation is valid only when the phase induced by the specimen is less than π/2 (Lim *et al.* 2015; Slaney *et al.* 1984). For a single biological cell with a typical thickness of about 10 μm, and the RI difference with respect to the medium about 0.03 at wavelength λ = 632.8 nm, the phase induced by the cell is 0.95π, larger than π/2. Therefore, the Born approximation is not expected to be accurate for imaging thick biological cells.

In order to solve the above problem, a Rytov approximation model is proposed. Following Devaney’s method (Devaney 1981), if the RI variation of the weakly scattering object is much smaller than that of the surrounding medium *i.e.,* |*n*($\vec {\boldsymbol{r}} $)-*n*_m_|/*n*_m_ << 1, the scattering field, which is mainly caused by the perturbation of the RI, can be regarded as a small phase perturbation. The total field can be expressed in the form:

8 $ U(\vec {\boldsymbol{r}} ) = {U_i}(\vec {\boldsymbol{r}} ) + U_s^{}(\vec {\boldsymbol{r}} ) \approx {U_i}(\vec {\boldsymbol{r}} ){e^{U_s^{}(\vec {\boldsymbol{r}} )}} . $

Then, the scattered field can be calculated by ***U***_*s*_($\vec {\boldsymbol{r}} $)=ln[***U***($\vec {\boldsymbol{r}} $)/***U***_*i*_($\vec {\boldsymbol{r}} $)]. By substituting the 2D Fourier transform of ***U***_*s*_($\vec {\boldsymbol{r}} $) into the Eq. 6, and following the same reconstruction procedure outlined above, the quantitative 3D RI distribution of the scattering object can be calculated using Eq. 7. The valid condition of the Rytov approximation indicates that it is independent of the specimen size, but requires that the RI variation of the specimen is much smaller than the RI value of the surrounding medium. In practice, |*n*($\vec {\boldsymbol{r}} $) − *n*_m_| for biological cells typically falls in 0.03−0.05 (Sung *et al.* 2009), and the RI of the surrounding medium is larger than 1; thus, the Rytov approximation is generally satisfied. This means the reconstruction under the Rytov approximation is more accurate than the first-Born approximation.

It is worth mentioning that the conventional ODTM approach requires acquiring tens to hundreds of illumination-angle-varied scattering fields. The incident field, which propagates unperturbed in free space, must also be measured to calculate the complex phase (Kak *et al.* 2002). This is realized by capturing the projections of the measurement volume with no object present. In order to reduce the number of illumination angles, several numerical approaches have been proposed, including nonlinear forward models and regularization techniques (Bronstein *et al.* 2002; Kamilov *et al.* 2015, 2016; Sung and Dasari 2011). For instance, the beam propagation method combined with advanced regularization techniques, such as 3D total variation (TV) (Persson *et al.* 2001), offers an improved reconstruction of 3D RI.

## EXPERIMENTAL IMPLEMENTATION AND DATA PROCESSING

**Figure 3:**
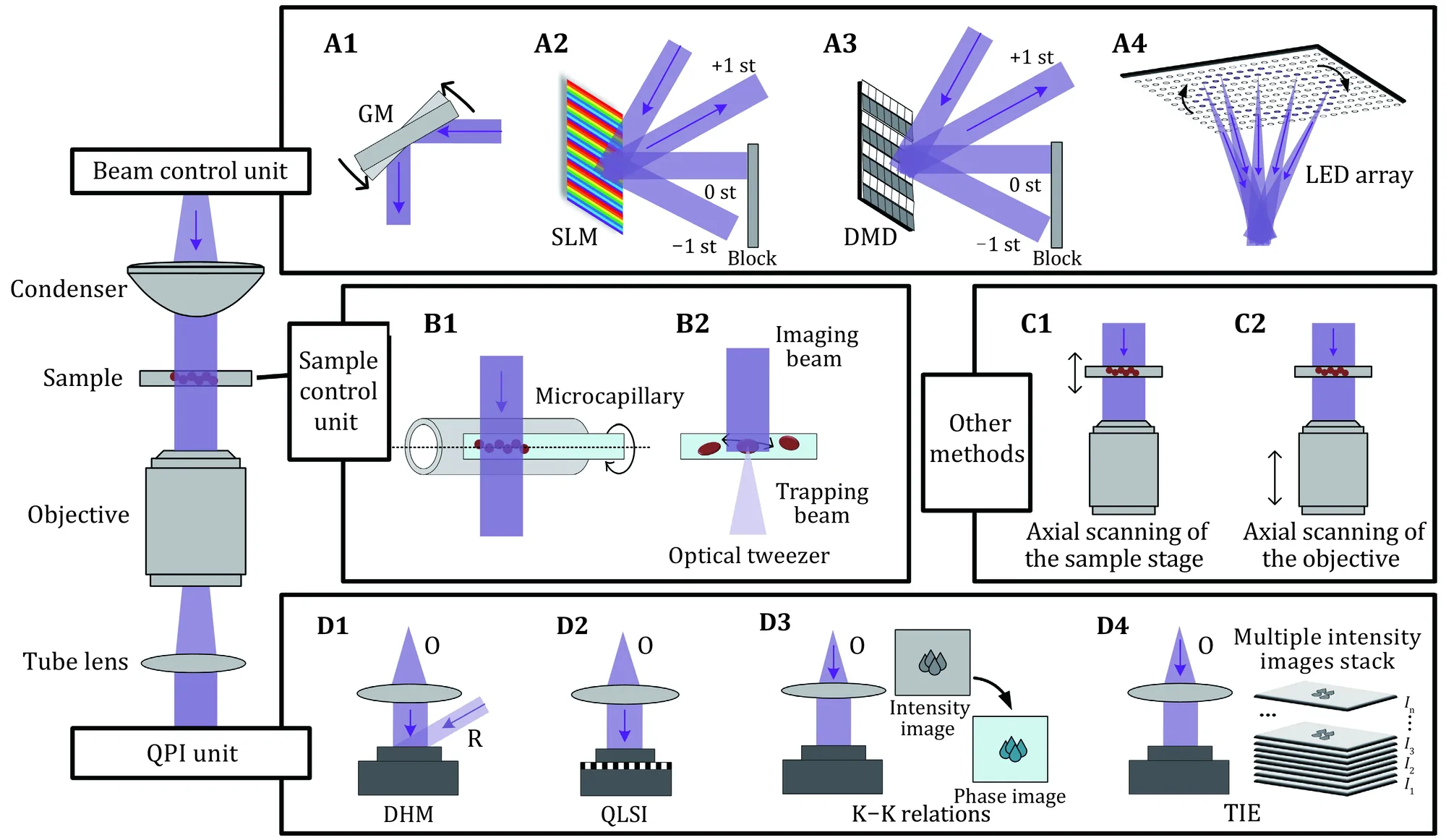
Fundamental implementation configuration of the transmission ODTM system. The GM (**A1**), SLM (**A2**), DMD (**A3**), programmable LED array (**A4**) can all be used to control the illumination angle. Rotating the sample using either microcapillary or in combination (**B1**) with optical tweezers (**B2**) is an alternative solution for ODTM. Axis scanning of the sample stage (**C1**) or objective (**C2**) is employed for ODTM. **D1**−**D4** Four typical QPI techniques used in ODTM. They are DHM, QLSI, phase retrieval based on Kramers−Kronig (K−K) relations, and TIE methods, respectively. O represents the object beam, and R represents the reference beam

According to the principle of ODTM, it is evident that the core of ODTM is accurately measuring the complex amplitudes of the specimen at various angles of illumination. Hence, ODTM systems must comprise two essential parts: one to control the angle of illumination incident on the sample, and the other is the QPI unit to measure the quantitative 2D optical fields of the specimen at each illumination angle. Figure 3 illustrates the fundamental implementation configuration of the transmission ODTM system. Coherent light is typically utilized as the illumination beam, which not only offers excellent directivity but also enables high-precision phase imaging. The collimated illumination light is modulated by the beam control unit and projected via the condenser system to the sample. The transmitted object beam **O** is then collected by the microscopic imaging system and enters the QPI unit, which measures the quantitative complex amplitude distribution at each angle of illumination. The illumination angle can be controlled by the beam control unit, including a GM (Kuś *et al.* 2019), SLM (Kuś *et al.* 2015), DMD (Shin *et al.* 2015), or programmable LED array (Li *et al.* 2017), as shown in Figs. 3A1−3A4 . Alternatively, rotating the sample using microcapillary (Dubois *et al.* 2006) (Fig. 3B1) or optical tweezers (Habaza *et al.* 2015) (Fig. 3B2) under a fixed illumination direction, integrated illumination rotation and specimen rotation (Kozacki *et al.* 2009), axis scanning the sample stage or objective (Lin *et al.* 2014) (Figs. 3C1 and 3C2), the wavelength scanning of an illumination beam (Yu and Kim 2005) can also be used to achieve ODTM. Recently, white-light illumination in combination with axial scanning was used for ODTM (Kim *et al.* 2014b). On the other hand, in order to obtain the quantitative 2D optical field at each illumination angle, various QPI methods have been employed, including interferometric methods (such as DHM and QLSI, Figs. 3D1 and 3D2) and non-interferometric methods (Baek and Park 2021; Gbur and Wolf 2002; Li *et al.* 2022; Liu *et al.* 2025; Shen *et al.* 2025; Zhu *et al.* 2022) (such as phase retrieval based on Kramers−Kronig (K−K) relations and TIE methods, Figs. 3D3 and 3D4). The emergence of intensity-based ODTM via K−K relations or TIE simplifies phase retrieval by replacing interferometric measurements with computational imaging, reducing hardware complexity while preserving reconstruction quality. However, the reconstruction algorithms often rely on the weak scattering assumption or the paraxial approximation, which degrade the phase measurement accuracy (de Groot 2015). High computational complexity due to the integral calculation in K−K relations and the 3D reconstruction process requires optimization for real-time imaging. Some non-interferometric approaches require multiple input images for phase reconstruction, which prolong the data acquisition time and thereby reduce the temporal resolution (Huang and Cao 2024) . Until now, the most popular configuration of the ODTM system is DHM with rotational illumination. This configuration has already been used in commercial products by NanoLive and TomoCube. These systems have been applied in dynamic cell analysis and integrated with microfluidics for high-throughput screening (Aharoni *et al.* 2025; Debailleul *et al.* 2008; Lee *et al.* 2024; Merola *et al.* 2017; Pirone *et al.* 2022). Since QPI technologies and their performance have been thoroughly analyzed and reported (Chaumet *et al.* 2024; Huang and Cao 2024; Nguyen *et al.* 2022), we will investigate the impact of different illumination modes and reconstruction algorithms on ODTM imaging.

### ODTM with illumination rotation

Illumination rotation (IR) is the most commonly used approach in ODTM because it is relatively easy to implement and minimizes the alteration of the specimen. At the beginning, GMs were used to control the angle of the illumination beam by tilting mirrors of the galvanometer located at the conjugate plane of the sample (Fig. 3A1). The utilization of the galvanometer leads to minimal optical power loss. However, it introduces mechanical instability issues, such as position jittering from electrical noise and positioning errors at high voltage levels due to the nonlinear response characteristics. Besides, the rotational surfaces for two independent axes cannot be exactly conjugated to the sample plane due to the geometry of the dual-axis galvanometer, which introduces unwanted additional phase distribution into the illumination beam. To overcome this problem, placing two single-axis galvanometers at separate conjugate planes relayed by additional 4*f* lenses can be used, but the bulky configuration may cause additional phase instability.

With the development of light field modulation technologies and devices, SLM and DMD are used as beam controllers (Kuś *et al.* 2015; Shin *et al.* 2015). They are located at the conjugate plane to the sample. Plane waves with desired propagating directions can be generated after displaying holograms or binary patterns on them. The unwanted diffracted beams should be blocked as shown in Figs.3A2 and 3A3. Since the SLM and DMD do not contain mechanically moving components, the ODTM with SLM/DMD can be performed with high stability (Shin *et al.* 2015; Zheng *et al.* 2024). Moreover, the SLM and DMD can correct the wavefront distortion of the illumination beam to generate clear illumination plane waves at the sample plane, which enhances the accuracy of ODTM. Furthermore, one can use SLM to create self-accelerating Airy beams and steer Airy beams into various accelerating orientations, generating views of the object from a full set of perspectives to realize Airy-beam tomographic microscopy (ATM) (Wang *et al.* 2020). Nevertheless, due to the limited diffraction efficiency and the slow innate response of liquid crystal, SLM-based ODTM has a limited intensity utilization efficiency and scanning rate. By contrast, DMD enables a fast refreshing rate but is difficult to generate arbitrary illumination directions due to its pure intensity modulation feature.

In addition, the programmable LED arrays can also serve as the light source for ODTM, enabling multi-angle illumination of the sample. The light emitted from a quasi-monochromatic or chromatic LED array (Zhou *et al.* 2022a) illuminates the specimen from a far distance in a specific direction, as shown in Fig. 3A4. The operation of the programmable LED array is simple, and it has a relatively low cost compared to GM, SLM, and DMD. The light beam emitted by the LED is partially coherent, which effectively reduces the speckle noise and improves the imaging quality but at the cost of decreased accuracy. Moreover, the subsequent QPI method should be guaranteed to work properly under the partially coherent light illumination.

**Figure 4:**
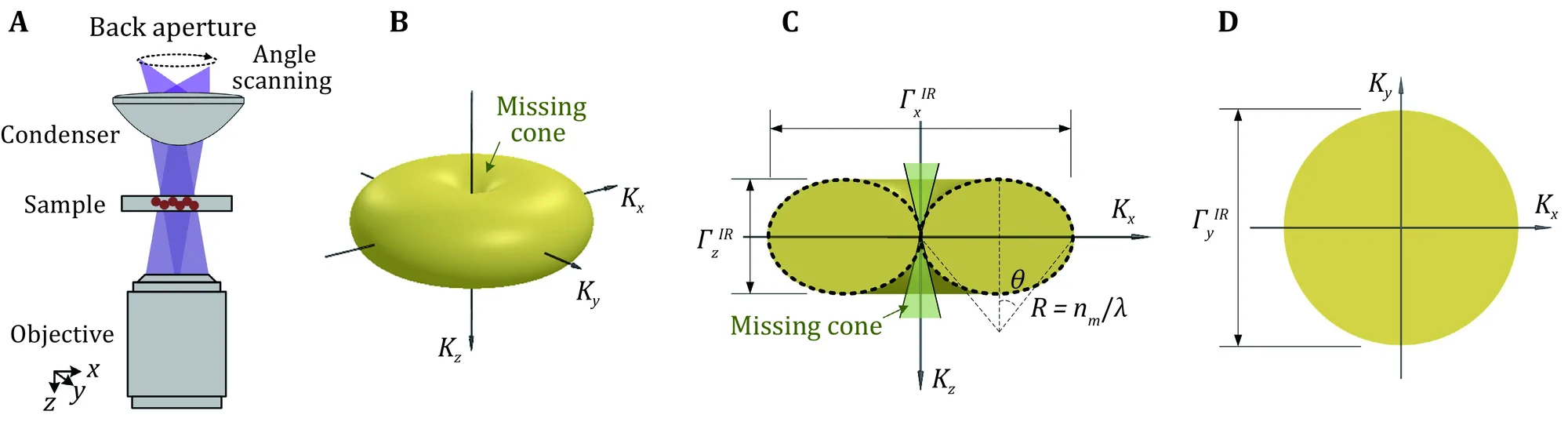
ODTM with illumination rotation. **A** Typical configuration. **B** Obtained 3D OTF. **C**,**D** Cross-sectional slices of the OTF in *K*_*x*_−*Kz*_*z*_ and *K*_*x*_−*K*_*y*_ planes, respectively

The performance of the ODTM system with the illumination rotation scheme is analyzed below. As described in the previous section, only a portion of the Ewald sphere is effectively collected under a single illumination incident angle *θ*_*i*_. The maximum illumination angle *θ* is equal to the aperture angle of the imaging objective lens. When a large number of incident waves with varying angles sequentially illuminate, a volume of the Ewald spheres is obtained to form the system’s optical transfer function (OTF), as depicted in Fig. 4B. The doughnut shaped volume is equally generated by the rotation of the section in Fig. 4C around the optical axis. A detailed explanation of the construction can be found in references (Debailleul *et al.* 2008; Vinoth *et al.* 2018). Notice that there is a missing spatial frequency region in the *K*z axis, known as the “missing cone”, which is caused by the finite *NA* of the imaging objective. The frequency support for the ODTM with illumination rotation is shown in Figs. 4B and 4D, and the formula is given by Eq. 9 (Lauer 2002).

9 $ \varGamma _x^{IR} = \varGamma _y^{IR} = \frac{{4{n_{\mathrm{m}}}\sin \theta }}{\lambda }\;,\;\;\;\varGamma _z^{IR} = \frac{{2{n_m}(1 - \cos \theta )}}{\lambda } \; . $

The ODTM with illumination rotation offers the advantage of rapid illumination switching, rendering the observation of dynamic phenomena within living specimens (Debailleul *et al.* 2009). However, a notable drawback is the anisotropic resolution, characterized by a lower resolution along the optical axis compared to that in the lateral plane. This issue can be partially solved by other imaging configurations and reconstruction algorithms.

### ODTM with specimen rotation

**Figure 5:**
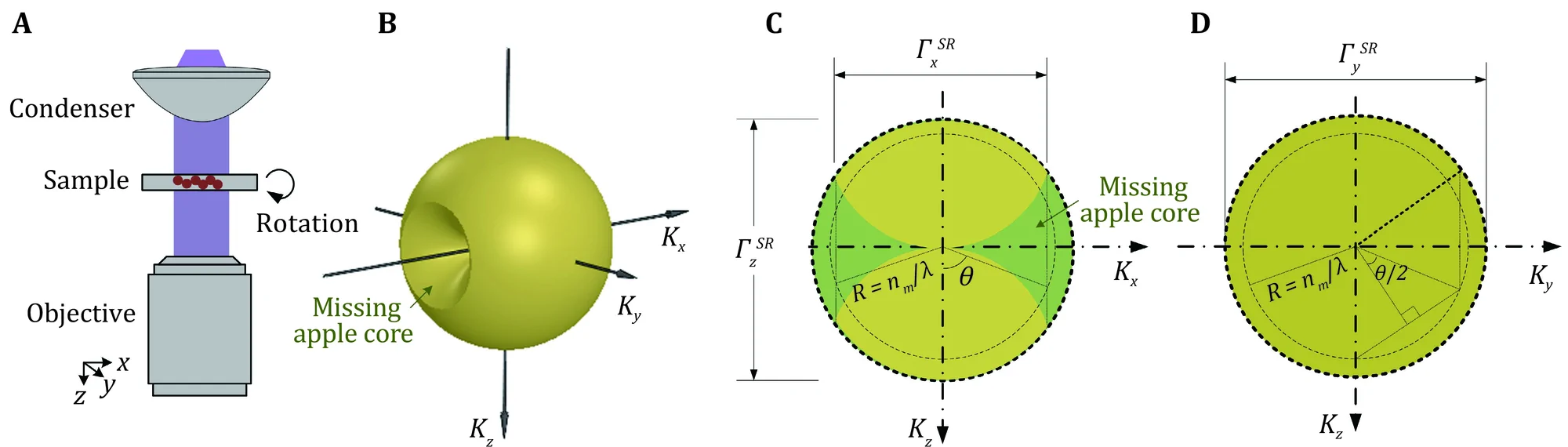
ODTM with sample rotation typical configuration (**A**), obtained 3D OTF (**B**). **C**,**D** Cross-sectional slices of the OTF in the *K*_*x*_−*K*_*z*_ and *K*_*y*_−*K*_*z*_ planes, respectively

Different from the illumination rotation mode, the specimen rotation (SR) mode has a whole range of viewing angles. The spectral components in directions perpendicular to the rotational axis are nearly fully obtained. But a small subset of uncaptured frequencies remain along the rotational axis (here is the *x*-axis) due to the curvature of the successive caps of the Ewald sphere, which is the so-called “missing apple core” (Vertu *et al.* 2009). The frequency support for the ODTM with specimen rotation, as shown in Figs. 5C and 5D, is given as Eq. 10.

10 $ \varGamma _{\mathrm{y}}^{SR} = \varGamma _{\mathrm{z}}^{SR} = \frac{{4{n_{\mathrm{m}}}\sin (\theta /2)}}{\lambda } \;,\;\;\;\; \varGamma _x^{SR} = \frac{{2{n_{\mathrm{m}}}\sin \theta }}{\lambda } \; . $

Rotating the specimen is usually performed by embedding the sample within a rotary micropipette or a capillary (Kuś *et al.* 2014; Lin and Cheng 2014). A very thin syringe needle (Sullivan and McLeod 2007), electric field (Le Saux *et al.* 2009), optical tweezers (Memmolo *et al.* 2014; Müller *et al.* 2015) were also utilized to rotate the sample. However, the rotation of samples restricts the data acquisition speed, and also may cause deformation of live cells. It is not suitable for measuring adherent cells, as these cells are incapable of being rotated.

### ODTM with other methods

**Figure 6:**
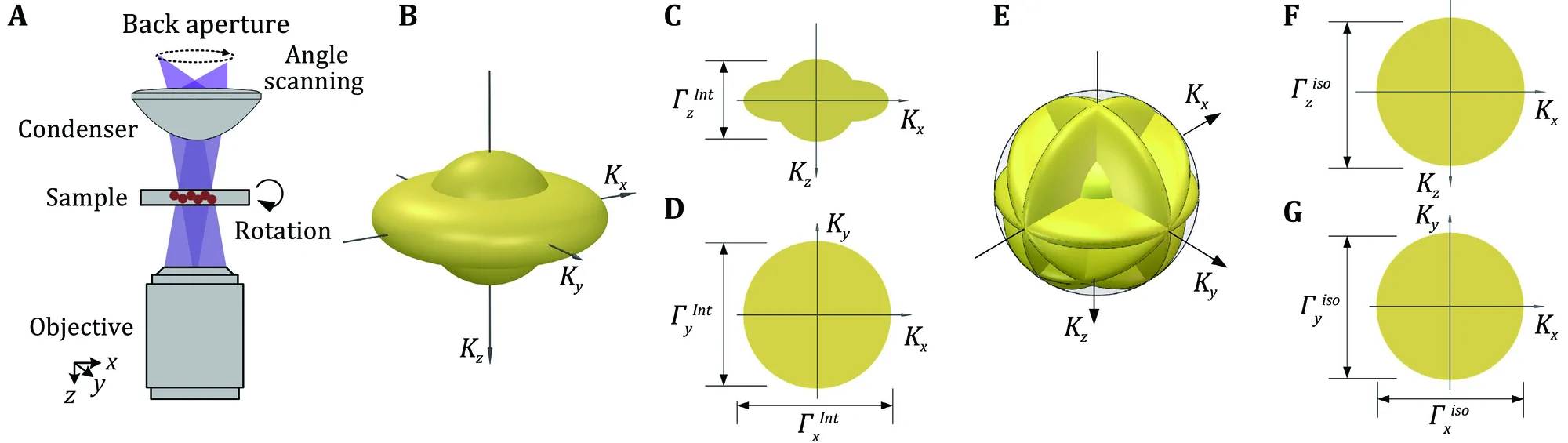
ODTM with integrated illumination rotation and sample rotation. **A** Typical configuration. **B** Obtained 3D OTF of ODTM with full-angle sample rotation and a circular beam rotation. **C**,**D** Cross-sectional slices of Panel B in *K*_*x*_−*K*_*z*_ and *K*_*x*_−*K*_*y*_ planes, respectively. **E** Obtained 3D OTF of ODTM with specimen rotation and illumination rotation in a multiplexing mode. **F**,**G** Cross-sectional slices of Panel E in *K*_*x*_−*K*_*z*_ and *K*_*x*_−*K*_*y*_ planes, respectively

It is seen that the missing cone is aligned along the optical axis of the system, whereas the missing apple core is oriented along the specimen rotational axis, typically perpendicular to the optical axis. Therefore, one can combine both approaches to achieve isotropic resolution images (Lee *et al.* 2021; Simon *et al.* 2017; Vertu *et al.* 2011). Nevertheless, different combination approaches will produce different effects, as shown in Fig. 6. If one combines a full-angle sample rotation and a circular beam rotation, *i.e.*, combining the “doughnut” with the “ball” directly, then the obtained OTF exhibits a shape of an unidentified flying object, as shown in Fig. 6B. The center slice images show extended spatial frequency coverages in Figs. 6C and 6D, respectively. The supported frequencies are given as Eq. 11.

11 $ \varGamma _x^{Int} = \varGamma _y^{Int} = \frac{{4{n_{\mathrm{m}}}\sin \theta }}{\lambda }\;\ ,\;\;\;\; \varGamma _z^{Int} = \frac{{2{n_m}\sin \theta }}{\lambda }\; . $

If one combines the specimen rotation and the illumination rotation in a multiplexing mode, *i.e*., at each specimen rotation angle, performing the full illumination rotation, then the resulting OTF will exhibit a larger ball with a radius equivalent to that of the doughnut, as shown in Fig. 6E. The frequency supports are shown as Eq. 12.

12 $ \varGamma _x^{iso} = \varGamma _y^{iso} = \varGamma _z^{iso} = \frac{{4{n_{\mathrm{m}}}\sin \theta }}{\lambda }\; . $

The resolution in this case will be the same in all directions. Of course, the entire recording process is time-consuming due to the multiple rotations of the sample and illumination, which also demands high system stability.

Alternatively, axial scanning of either the objective or sample stage was also employed for ODTM. These methods can simplify optical setups, but have limited axial resolution. The use of low-coherence illumination sources and deconvolution algorithms have been reported for better axial resolution (Bon *et al.* 2014; Kim *et al.* 2014a; Phillips *et al.* 2012). However, the acquisition rate is reduced because the speed of the linear moving mechanism is slower than that of the GM, SLMs, and DMDs. Besides, scanning wavelengths of the illumination beam were also applied for ODTM (Huang *et al.* 2024; Liu *et al.* 2022b; Ossowski *et al.* 2022). But the wavelength change itself is insufficient to provide enough frequency coverage and preferably requires additional illumination rotation (Jung *et al.* 2016a). Recently, light-field microscopy, lens-free holographic Ptychography, and QLSI techniques have been introduced into the ODTM system to simplify the system configuration (Horstmeyer *et al.* 2016; Wu *et al.* 2024; Xie *et al.* 2024; Xiong *et al.* 2021; Zhou *et al.* 2022b).

### Reconstruction procedures for ODTM

The ODTM reconstructs the 3D RI map of an object from measurements of multiple 2D complex amplitudes at various illumination angles. The reconstruction algorithm is an important process of determining the spatial resolution and quantification of the complex RI. In the early stage, the researcher usually used beam projection reconstruction algorithms, such as the filtered back-projection algorithm (Mangal and Ramesh 1999) and Fourier mapping algorithm (Guo and Devaney 2005), to reconstruction the tomographic imaging of the specimen as these algorithms are maturely applied in computed tomography. They assume that the illumination beam propagates in a straight path, and the light diffraction inside the samples is ignored. In this case, the 2D Fourier spectrum of the measured optical field at a certain illumination angle is identical to the inclined planar slice of the 3D Fourier spectra of the object. The 3D RI distributions of optical elements and individual cell samples can be successfully measured with these algorithms. However, these linear algorithms are only valid when the size of the specimens is much bigger than the wavelength of illumination. Otherwise, the use of these algorithms will result in distorted shapes and incorrect RI values (Kamilov *et al.* 2016).

**Figure 7:**
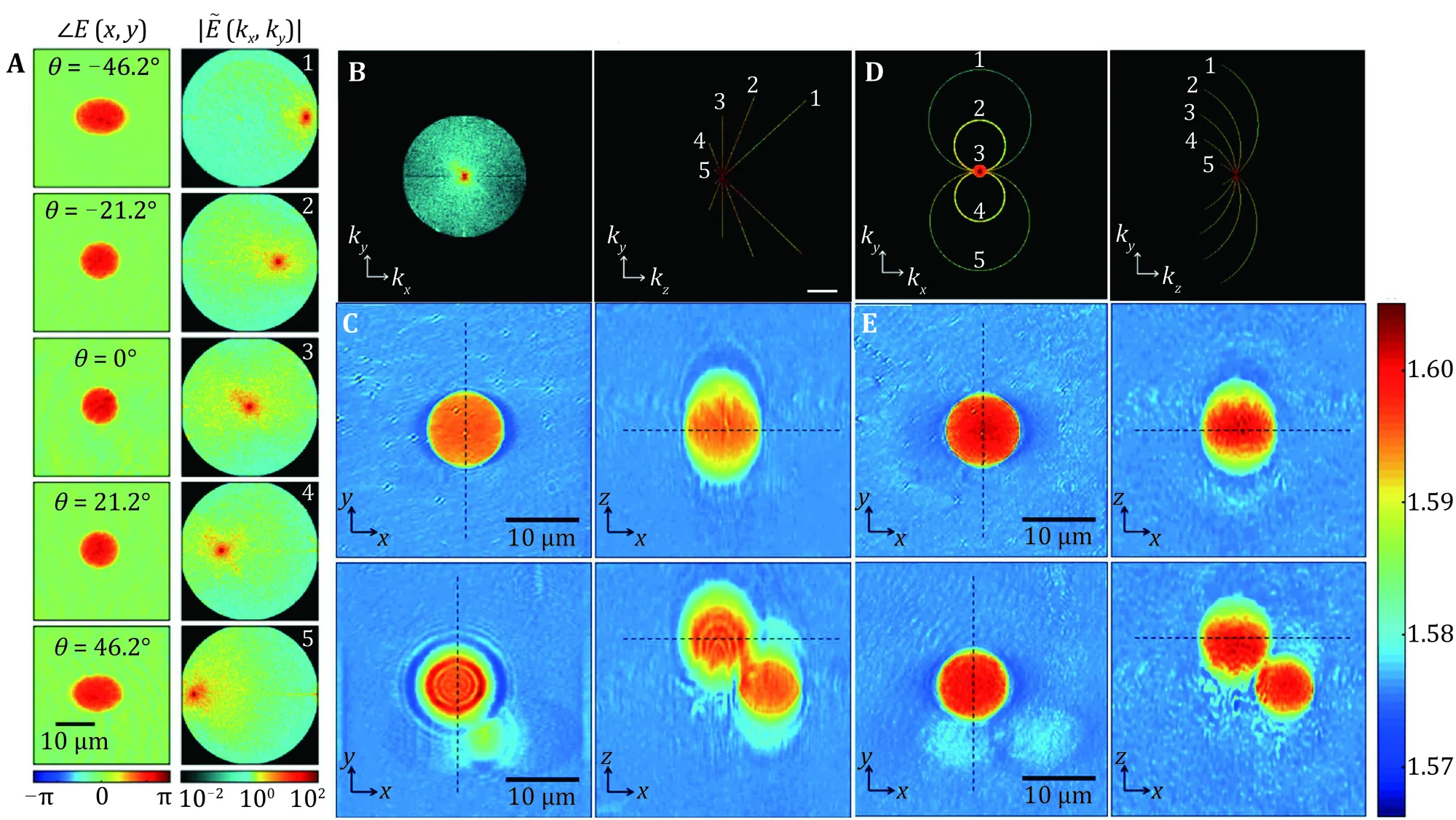
Comparison between the optical projection and diffraction reconstruction algorithms. **A** Quantitative phase images of the PS bead at various incidence angles, and the corresponding 2D Fourier spectra. OTF mapped by projection reconstruction algorithms (**B**) and diffraction reconstruction algorithm (**D**). **C**,**E** Sections of the reconstructed 3D RI distribution by projection and diffraction reconstruction algorithms, respectively. Adapted from Ref. Kim *et al.* (2016b)

Since the diffraction reconstruction algorithms, such as the Gerchberg-Papoulis (GP) algorithm (Papoulis 1975), are based on solving the Helmholtz equation for the incident and scattered optical fields, they can reconstruct more precise shapes and RI values than the projection algorithms. According to the Fourier diffraction theorem, the 2D Fourier spectra of the measured optical field at a certain illumination angle are mapped onto the surface of the Ewald sphere. The center position of the Ewald sphere is translated from the origin in the 3D Fourier space by a distance and direction corresponding to the vector of the incident angle. Figure 7 shows the results of a comparison between the projection reconstruction algorithm and the diffraction reconstruction algorithm on a polystyrene (PS) bead with a diameter of 10 μm with various illumination angles. One can clearly find that the diffraction reconstruction algorithm achieves more precise RI values and shapes of samples. Moreover, nonlinear high-order scattering models explicitly account for the complex multiple scattering of light within the sample, such as the wave propagation method (Suski *et al.* 2020), the multilayer Born method (Chen *et al.* 2020), the Lippmann−Schwinger equation-based method (Pham *et al.* 2020), the Born series method (Lee *et al.* 2022b) and the beam propagation method (Ali *et al.* 2025). These methods substantially improve the reconstruction fidelity and accuracy, but at the cost of considerably higher computational complexity.

The traditional ODTM reconstruction techniques rely on a formal definition of the forward scattering model and are subject to the limitations of a rigid mathematical construct, including boundary artifacts and scattering approximations resulting in phase biases, low-frequency blurring, and reduced axial resolution. Owing to the significant development in the field of electronics, such as digital image sensors and graphic processing units, artificial intelligence (AI) and deep learning (DL) have been developed rapidly, opening new avenues for ODTM (Yoon *et al.* 2017). Instead of being explicitly programmed or rule-based, AI-based reconstruction algorithms optimize adjustable parameters through data-driven learning with deep neural networks, demonstrating remarkable performance across various disciplines, particularly in handling large-scale, high-dimensional data. Consequently, numerous DL-based reconstruction algorithms have been reported (Kamilov *et al.* 2015; LaRoque *et al.* 2008; Liu *et al.* 2024, 2025; Saba *et al.* 2022; Sun *et al.* 2018; Yang *et al.* 2020, 2023), and they are realized by training a network that learns the underlying forward/inverse operation of a given optical system or specific image-to-image transformation. Once the network is optimally trained, it can rapidly perform an image inference task without any iterations or additional parameter tuning, and quasi-real time 3D imaging is achievable. Furthermore, the missing-cone and the missing apple core problems are partially overcome by including prior knowledge such as total variation (TV) (Krauze *et al.* 2016), non-negativity constraints (Lantéri *et al.* 2001), sparsity-enforcing penalties (Papoulis 1975), plug-and-play priors (Kamilov *et al.* 2017). A comprehensive analysis of the latest deep-learning advances in ODTM can be found in reference (Guo *et al.* 2025). Although each network has its unique advantages, there is no one network that can be universal. The generalization of the AI reconstruction algorithm needs to be improved, particularly as the reconstruction process still requires rigorous validation.

## APPLICATIONS OF ODTM IN BIOLOGICAL RESEARCH

Label-free 3D visualization of live cells has potentials in the investigation of functions and mechanisms of cells at the individual level. The ODTM enables label-free imaging of 3D refractive index distribution, which has emerged as one of the most powerful imaging tools for the study of cells and tissues in a noninvasive manner. The continuous development of innovative solutions also brings improved measurement accuracy, ease and certainty of analysis, as well as access to new functionalities and multimodality. The combination of sensitivity to activation biomarkers, dynamic monitoring capability, and clinical-grade throughput positions ODTM as a transformative tool for fundamental immunology research and therapeutic monitoring. This section reviews a few of the representative pathophysiological studies of cells, tissues and small-scale biological objects with the ODTM technique.

### Quantitative physiological parameters of single cells

Microscopic images reconstructed by ODTM can quantitatively and noninvasively retrieve the 3D RI distribution of cells and various physiological parameters of specimens, including morphological parameters (shape, volume, sphericity) and biophysical parameters (dry mass, dry mass density) can be further extracted from the quantitative RI information (Möckel *et al.* 2024). The retrieved structural, chemical, and mechanical information of cells can serve as important indicators for cell diagnosis and research.

**Figure 8:**
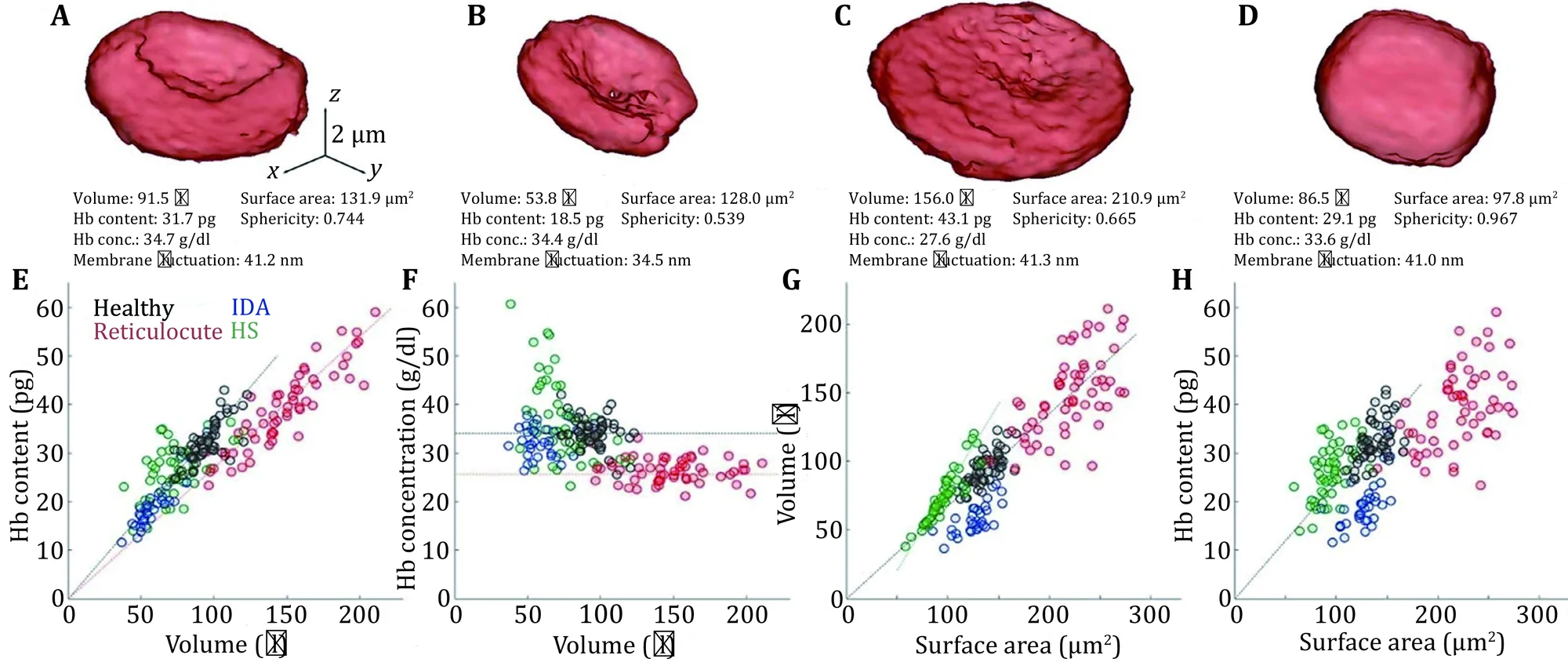
Investigation of RBCs with ODTM. **A**−**D** 3D rendered isosurfaces of RI maps of individual RBCs from healthy, IDA, reticulocyte, and HS groups, respectively. **E**−**H** Correlation maps between the morphological and the chemical parameter (Hb) for the four group RBCs. Adapted from Ref. Suresh (2006)

Red blood cells (RBCs) are one of the most widely studied biological specimens because of their important functions in living organisms: carrying oxygen from the lungs to tissues. The morphological, chemical, and mechanical parameters of RBCs have strong correlations with the pathophysiology of many diseases (Suresh 2006). Therefore, measuring the morphological, chemical, and mechanical properties of individual RBCs is essential to understanding the pathophysiology of a number of diseases in hematology. It may open up a new possibility for diagnosing diseases in the early stage. By using the ODTM technique, the researchers can simultaneously measure multiple parameters of RBCs (Kim *et al.* 2014c; Lee *et al.* 2017). The chemical composition of RBCs, particularly cytoplasmic hemoglobin (Hb), is used for medical diagnosis on a daily basis in laboratory medicine. Figures 8A−8D show the 3D RI tomograms of individual RBCs with four different pathophysiological groups: from a healthy individual (A), from a patient with iron deficiency anemia (IDA) (B), from a patient with a high reticulocyte count (C), and from a patient diagnosed with hereditary spherocytosis (HS) (D). The reconstructed morphologies exhibit good agreement with the known reference information. The RI tomograms of RBCs clearly exhibit that the RBCs from different groups have different shapes: RBCs from the healthy individual exhibit the characteristics of biconcave shape, whereas those from the patient with IDA exhibit a decrease in the cell volume. The RBCs from the patient with high reticulocyte content display a significant increase in mean volume, and those from the patient diagnosed with HS exhibit a spherocytosis shape. Further correlation analyses between the morphological and chemical parameters of the four groups are presented in Figs. 8E−8H. The correlations between the red cell indices demonstrate that they could be classified into different pathophysiological types. ODTM can be applied to a wide range of cell types, including but not limited to white blood cells, circulating tumor cells, bacterial cells, and endothelial cells, for biophysical and medical analysis and diagnosis.

**Figure 9:**
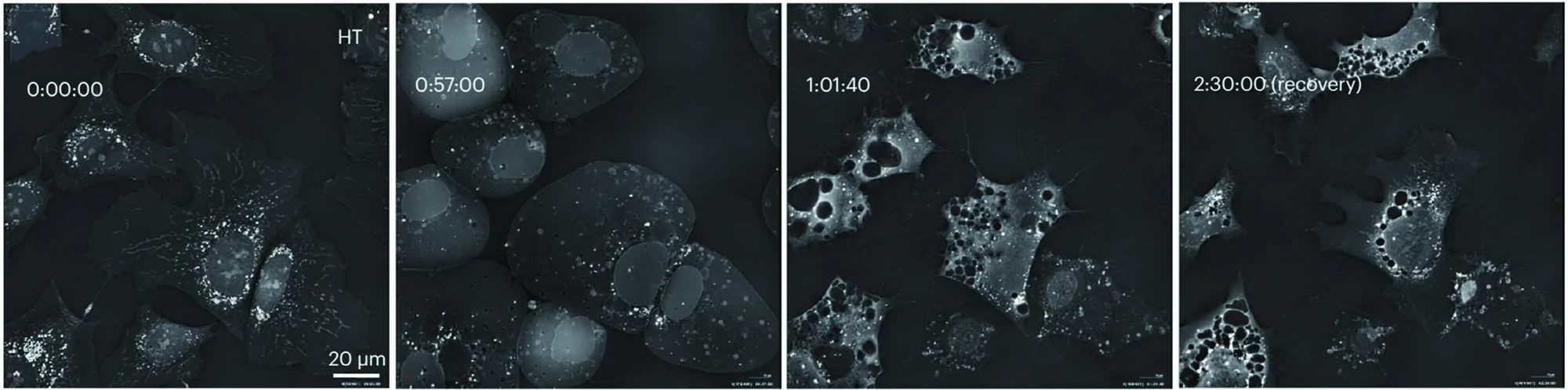
Time-lapse observation of the live Hep3B cells. Adapted from Ref. Kim *et al.* (2024a)

Because of its non-invasive and label-free visualization, ODTM plays an increasingly important role in live cell imaging for the study of cell growth, division, and differentiation (Karandikar *et al.* 2019; Kim *et al.* 2014b, 2021, 2024a; Sung *et al.* 2009). By tracking the changes in refractive index over time, researchers can monitor the progression of these processes and gain a better understanding of the underlying mechanisms. For instance, ODTM can effectively discriminate non-activated subtypes (B, CD4+/CD8+ T cells) through 3D refractive index tomography and machine learning, while it particularly excels in detecting activated T cells by capturing their dramatic biophysical changes. Activated CD8+ T cells show a 63% increase in dry mass (53.8 ± 32.4 pg vs 33.1 ± 13.1 pg in naïve cells, *p* < 0.001) and distinct morphological alterations changes far more pronounced than the subtle differences between resting lymphocyte subsets. Moreover, ODTM has also been used to investigate the effects of drugs, toxins, and other external stimuli on cells, providing a non-invasive and quantitative approach to drug screening and toxicity testing (Kim *et al.* 2019; Lee *et al.* 2020; Oh *et al.* 2020). Figure 9 presents the ODTM time-lapse observation of Hep3B cells, illustrating subcellular morphology changes upon H_2_O_2_ treatment, followed by cellular recovery after returning to the regular cell culture medium.

### Investigations of tissues and small-scale biological objects

**Figure 10:**
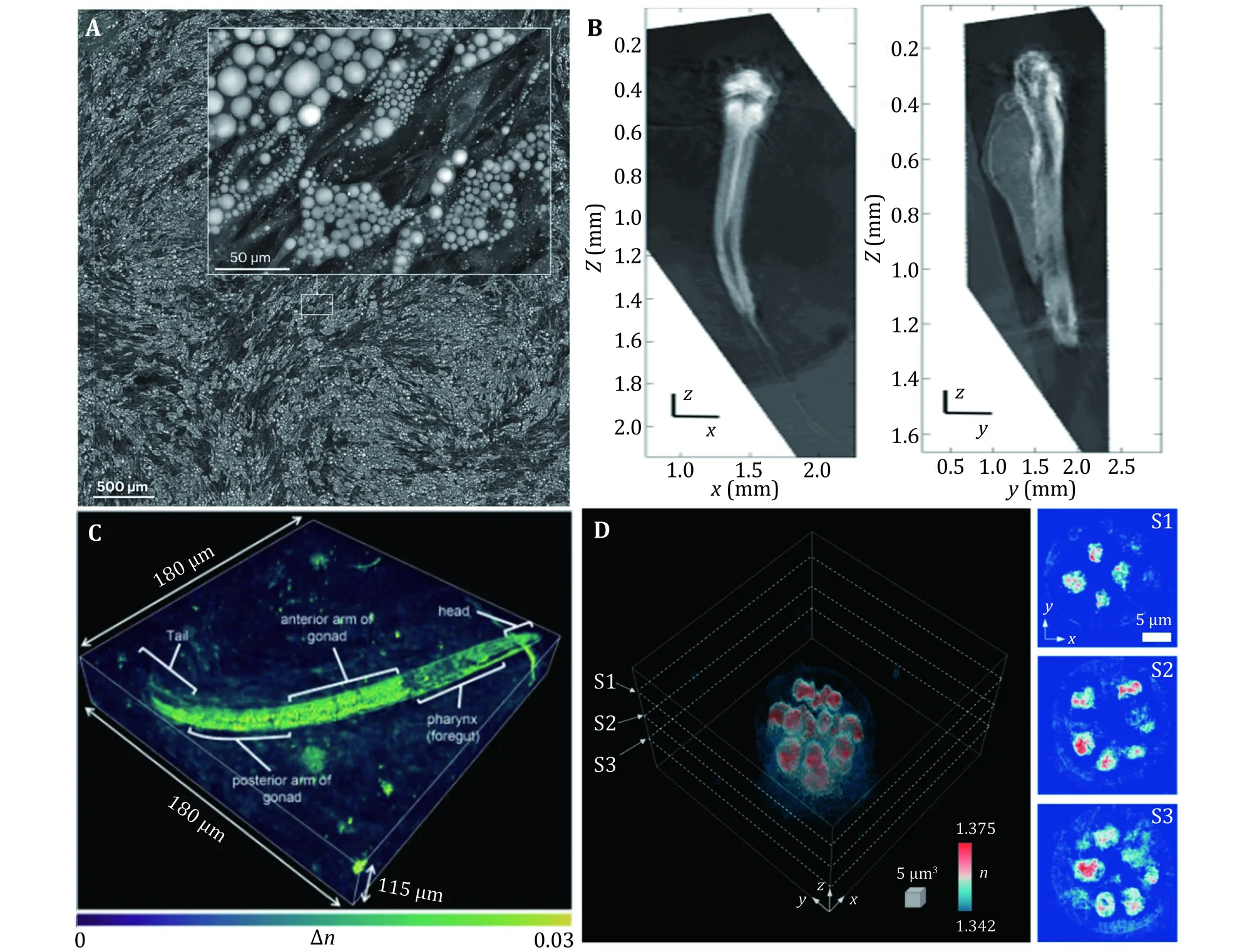
Investigations of the 4 mm × 4 mm stitched RI tomogram of fully differentiated adipocytes from the induced stem cells, with zoom-in images of adipocytes in the inset (Kim *et al.* 2024a) (**A**), zebrafish larvae (van Rooij and Kalkman 2019) (**B**), *C. elegans* (Li *et al.* 2022) (**C**) and *E. elegans* (Yuan *et al*. 2024) (**D**) with ODTMs

Since the objective lenses with high numerical aperture are usually adapted in ODTM for high-resolution imaging, and thus the field of view is limited to 100−200 μm (Kim *et al.* 2024a). In order to expand the field of view, one can measure multiple fields of view and stitching them after reconstructions. Figure 10A shows an example of the 4 mm × 4 mm stitched two-dimensional RI distribution image of adipocytes differentiated from fibrocytes. In addition to cellular observation and measurement, ODTM is also capable of visualizing various tissues and small biological specimens, including 3D histopathology, cancer tissue classification, and utilizing RI as a biomarker of diseases in tissue biopsies (Kim *et al.* 2022b, 2024b; van Rooij and Kalkman 2019; Wang *et al.* 2011b). Researchers can examine the intricate internal structures of these tiny creatures without the need for invasive procedures or harsh chemical treatments, preserving the integrity of the samples. This non-invasive approach is particularly beneficial for studying rare or sensitive specimens.

Zebrafish larvae have been widely used as models to gain insight into human disease. An optically cleared zebrafish larva in 13 mm^3^ volume was successfully investigated using ODTM with an isotropic resolution of 4 µm, as shown in Fig. 10B. The researchers demonstrated a clinical application of the technique by imaging an entire adult cryoinjured zebrafish heart. Besides the zebrafish larva, the 3D RI tomograms of the juvenile *C. elegans* worm (Li *et al.* 2022) over a volume of 180 µm × 180 µm × 115 µm and an *E. elegans* (Yuan *et al*. 2024) were also obtained with ODTM, as shown in Figs. 10C and 10D, respectively. The difference in RI rendering by colors of the glycoprotein matrix in *C. elegans* vividly represents its morphology. For the *E. elegans,* the cells within the colonies are distributed throughout the colloid in a specific pattern, which is visible in different *x*−*y* cross sections as depicted in Fig. 10D, denoted by “S1”, “S2” and “S3” for different depths. It can be seen that there are four cells in the top layer, and six cells in the middle and bottom layers, respectively. Interestingly, the cells in each layer are almost evenly distributed in a circle.

**Figure 11:**
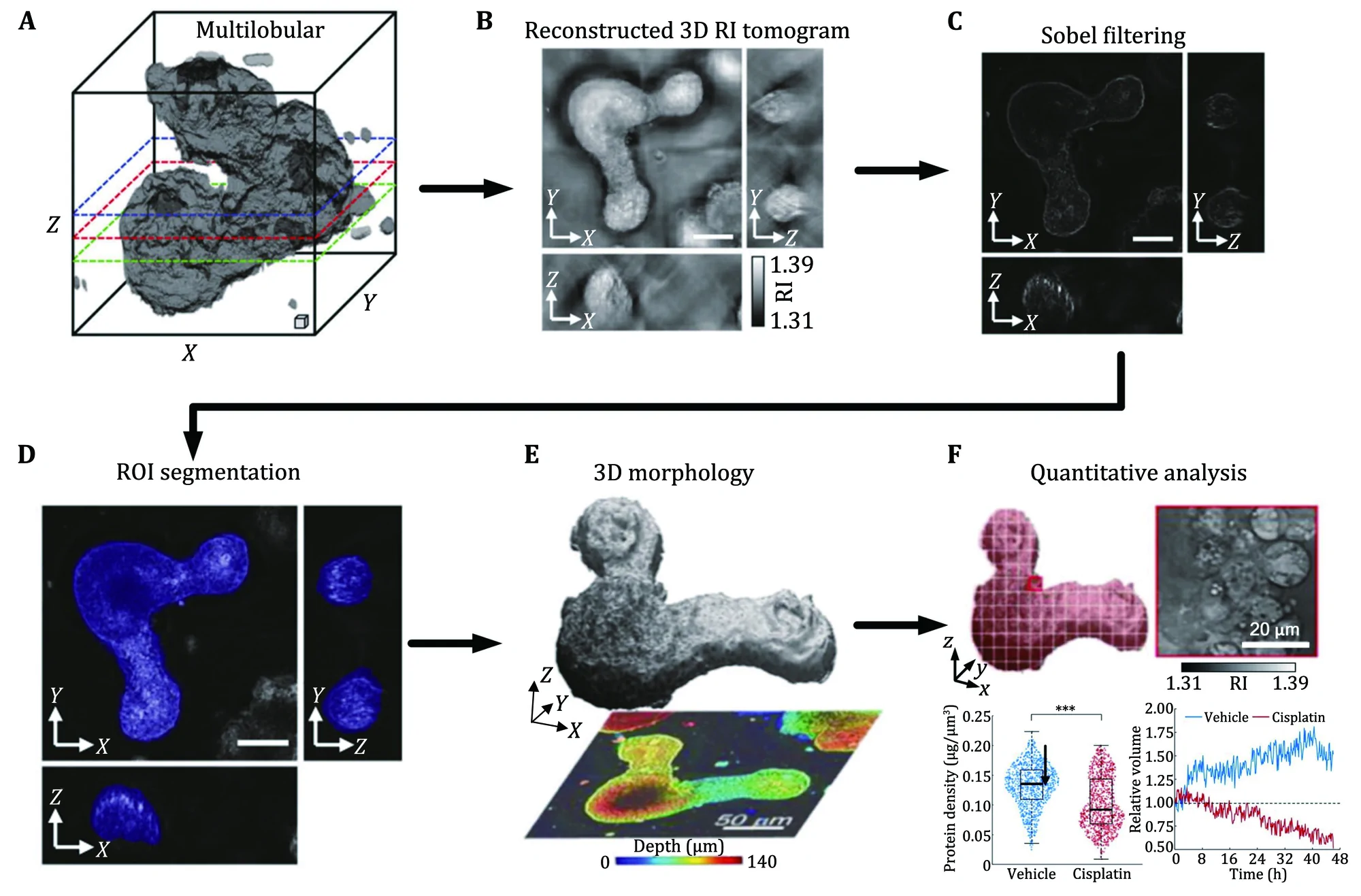
Workflow of segmentation and quantitative analysis of live mouse small intestinal organoids (sIOs) with ODTM. **A** The 3D rendered image of multilobular sIOs, scale boxes represent a volume of 10 µm × 10 µm × 10µm (width, height, and depth, respectively. **B** Reconstructed 3D RI tomogram. **C** Generated images with clearly delineated boundaries after Sobel filtering. **D**−**F** Longitudinal quantitative assessment of volume. Adapted from Ref. Lee *et al.* (2024)

Beyond morphology, extracting biologically meaningful parameters from reconstructed 3D quantitative RI through imaging segmentation and quantitative analysis is essential. Imaging segmentation in ODTM typically involves preprocessing to enhance boundaries and distinguish target structures from the background. For instance, a Sobel filter is applied to gradient images to mitigate aberration-induced artifacts, clarifying organoid or cellular outlines (Lee *et al.* 2024). Interactive machine learning tools like ilastik are then used for precise segmentation, enabling the labeling of specific regions of interest (ROI) (Berg *et al.* 2019). From segmented ROIs, RI values are converted to quantitative metrics including volume, surface area, sphericity, protein density, total mass *etc.* to characterize morphological features and assess cellular composition (Hong *et al.* 2021). Figure 11 provides an overview of the workflow for segmentation and quantitative analysis of live mouse small intestinal organoids (sIOs) using ODTM. Figures 11A and 11B show the 3D rendered image of multilobular sIOs within a 10 µm × 10 µm × 10 µm volume and the reconstructed 3D RI tomogram from ODTM, respectively. A Sobel filter is then employed to delineate clear boundaries of the specimen. Further segmentation and labeling of organoid outlines, as shown in Fig. 11D, are accomplished using the open-source user-interactive segmentation toolkit ilastik. This allows for the intuitive visualization of the organoid’s spatial structure, including crypt branching and lumen distribution, which better reflect their real structural characteristics compared to 2D views, as shown in Fig. 11E. At last, key parameters such as volume, protein density, and lumen depth can be quantitatively analyzed over depth or time, as shown Fig. 11F. These segmentation and quantitative approaches highlight ODTM as a powerful tool for label-free, high-resolution analysis of complex biological systems, providing data support for studying organoid growth kinetics or their responses to drugs (Kim *et al.* 2022a).

It should also be noted that there remains a challenge for ODTM in the implementation of thick tissues and specimens due to the intense light scattering. In such scenarios, the RI variation of tissues and biological objects from the surrounding medium is too large to minimize the refraction, diffraction, and scattering effects. Consequently, the first-Born approximation and the Rytov approximation are no longer valid. One of the solutions is using the tissue clearing technique (Richardson and Lichtman 2015), which enables biological tissues to be transparent, allowing for high resolution imaging of entire tissue samples. This approach aligns with the pursuit of overcoming optical barriers in thick specimen imaging, as ODTM’s capacity to capture fine structural details relies heavily on minimizing light scattering — a challenge that tissue clearing directly mitigates by reducing optical inhomogeneities within thick tissues.

## SUMMARY AND PERSPECTIVES

As a powerful and promising label-free quantitative 3D imaging and analysis technique, ODTM has been increasingly employed in the fields of biology and promises to open numerous unexplored applications. Here, we reviewed the principle of ODTM, introduced the instrumental requirements, and summarized the different illumination modes and reconstruction algorithms. The illumination rotation schemes using GM, DMD, SLM, or programmable LED arrays demonstrate merits in the practical application of ODTM. These schemes are easy to be implemented, minimize alteration of the specimen, and offer fast imaging speed. But these schemes exhibit anisotropic spatial resolution along the optical axis and the transverse direction. The sample rotation scheme of ODTM obtains quasi-isotropic spatial resolution at the cost of limited data acquisition speed. The ODTM with integrated illumination and sample rotations can simultaneously achieve the highest isotropic spatial resolution, but requires a time-consuming recording process and a highly stable environment. With ODTM, researchers can observe biological specimens at both cellular and tissue levels. Measuring 3D RI maps of live cells plays an important role in understanding of cell pathophysiology.

As a new rising 3D optical imaging technique, the capability of ODTM can be further improved. ODTM with special optical design have been reported to enhance the performance and practical applications in biological and clinical settings (Tang *et al.* 2025; Verrier *et al.* 2024; Yang *et al.* 2024; Zhou *et al.* 2023, 2024). New reconstruction algorithms that rely on multi-layer models have been considered for the multiple-scattering thick samples and larger refractive index variation (Bazow *et al.* 2023; Dunn *et al.* 2024; Fan *et al.* 2020; Moser *et al.* 2023; Zhou *et al.* 2025). More and more intelligent data processing algorithms have been employed in ODTM with the rapid development of AI techniques. These approaches not only bring solutions to the common issue of “missing frequency” in 3D spectrum synthesis but also reconstruct the 3D RI distribution with less data, improving both the accuracy and efficiency for the observation of living biological specimens (Chung *et al.* 2021; Liu *et al.* 2022a; Ryu *et al.* 2021; Saba *et al.* 2022). Beyond reconstruction, deep learning also expands ODTM's biomedical utility, *e*.*g*., enabling automated cancer cell identification via label-free 3D refractive index analysis (Hong *et al.* 2024) and dynamic intracellular nanoparticle tracking for therapeutic research (Liu *et al.* 2024).

Nevertheless, the AI approaches still face to some challenges, including heavy reliance on scarce training data and inherent limitations from their "black-box" nature, such as data dependency, limited generalizability, and lack of physical interpretability. Physics-informed frameworks subsequently emerge, which integrate optical principles (*e*.*g*., CNN-implicit priors or Physics-Informed Neural Networks enforcing Helmholtz equation constraints) to improve fidelity with less data (Saba *et al.* 2022; Zhou and Horstmeyer 2020). Crucially, given the inherent "black-box" nature of neural networks and their potential deployment in diagnostic or regulatory applications, rigorous validation of output data is imperative (Matlock *et al.* 2021).

Furthermore, advancements in polarization-diverse illumination and detection have driven the development of birefringent/polarization sensitive ODTM, enhancing sensitivity and selectivity (Li *et al.* 2025; van Rooij and Kalkman 2020; Saba *et al.* 2021; Song *et al.* 2023). By incorporating polarization-diverse measurements into the inverse problem, polarization-sensitive ODTM quantitatively reconstructs the three-dimensional distributions of both refractive index and the specimen's polarization response. Recent multislice vectorial models overcome the limitations of first-order Born and Rytov approximations, solving the full vector inverse scattering problem for highly birefringent media (Lee *et al.* 2022b; Mu *et al.* 2023; Zhou *et al.* 2025). Combined with structured illumination (Chowdhury *et al.* 2017; Liu *et al.* 2023), Raman spectroscopy (Anantha *et al.* 2023), or fluorescence assist (Dong *et al.* 2020), the ODTM can achieve sub-diffraction resolution molecular imaging.

Overall, ODTM has evolved into a versatile tool for 3D imaging of specimens, with applications spanning biology, materials science, and clinical research. Ongoing advancements are addressing challenges in resolution, speed, and depth, and future progress is likely to focus on AI integration, multimodal imaging, and translational clinical tools, solidifying its role in modern microscopy.
